# A Novel Insight into Screening for Antioxidant Peptides from Hazelnut Protein: Based on the Properties of Amino Acid Residues

**DOI:** 10.3390/antiox11010127

**Published:** 2022-01-06

**Authors:** Chenshan Shi, Miaomiao Liu, Hongfei Zhao, Zhaolin Lv, Lisong Liang, Bolin Zhang

**Affiliations:** 1 Beijing Key Laboratory of Forest Food Processing and Safety, College of Biological Sciences and Technology, Beijing Forestry University, Beijing 100083, China; shichenshan@bjfu.edu.cn (C.S.); 17853555780@163.com (M.L.); zhaohf518@163.com (H.Z.); zhaolinlv@bjfu.edu.cn (Z.L.); 2 Key Laboratory of Tree Breeding and Cultivation of the State Forestry and Grassland Administration, Research Institute of Forestry, Chinese Academy of Forestry, Beijing 100091, China; 3 State Key Laboratory of Tree Genetics and Breeding, Chinese Academy of Forestry, Beijing 100091, China; 4 Hazelnut Engineering and Technical Research Center of the State Forestry and Grassland Administration, Beijing 100091, China; 5 National Innovation Alliance of Hazelnut Industry, Beijing 100091, China

**Keywords:** antioxidant peptide, amino acid residues, hazelnut, BIOPEP database, DFT calculation

## Abstract

This study used the properties of amino acid residues to screen antioxidant peptides from hazelnut protein. It was confirmed that the type and position of amino acid residues, grand average of hydropathy, and molecular weight of a peptide could be comprehensively applied to obtain desirable antioxidants after analyzing the information of synthesized dipeptides and BIOPEP database. As a result, six peptides, FSEY, QIESW, SEGFEW, IDLGTTY, GEGFFEM, and NLNQCQRYM were identified from hazelnut protein hydrolysates with higher antioxidant capacity than reduced Glutathione (GSH) against linoleic acid oxidation. The peptides having Tyr residue at C-terminal were found to prohibit the oxidation of linoleic acid better than others. Among them, peptide FSEY inhibited the rancidity of hazelnut oil very well in an oil-in-water emulsion. Additionally, quantum chemical parameters proved Tyr-residue to act as the active site of FSEY are responsible for its antioxidation. This is the first presentation of a novel approach to excavating desired antioxidant peptides against lipid oxidation from hazelnut protein via the properties of amino acid residues.

## 1. Introduction

Peptides, consisting of amino acid residues, are now popular as antioxidants owing to their advantages related to absorption and safety [1]. The antioxidant activity is usually attributed to such properties as the amino acid composition, active amino acid position, molecular mass, and spatial structure of the peptides [1]. Regarding peptides, active amino acid residues, such as tyrosine (Tyr), tryptophan (Trp), cysteine (Cys), methionine (Met), and histidine(His) may act as hydrogen donors; acidic amino acid residues, such as aspartic acid (Asp) and glutamic acid (Glu) can chelate metal ion; hydrophobic amino acid residues, such as alanine (Ala), valine (Val), Proline (Pro), Phenylalanine (Phe) and leucine (Leu) may help to improve the solubility of peptides in the lipid phase, and facilitate interactions between the peptide and lipid-free radicals, thus increasing antioxidant activity [2]. At the same time, active amino acids Cys, Met, Trp, and Tyr, as well as peptides which are designed based on these residues, have been confirmed to eliminate reactive oxygen species (ROS), reactive nitrogen species (RNS), as well as ABTS (2,2-azino-bis-3-ethylbenzothiazoline-6-sulfonic acid) and DPPH (2,2’-diphenyl-1-picrylhydrazyl) radicals in real peptides’ system [1,3]. Thus, is it possible to directly screen antioxidant peptides according to the features of active amino acids? A survey of relevant literature by us has shown that few studies have been done in this aspect.

Currently, real-time updated protein databases, bioinformatics tools, and computer-aided mathematical models have become available for investigating the bioactive peptides. The databases BIOPEP, UniProt, and SwePep collect the physicochemical information of various protein-derived peptides for predicting their bioactivities from released protein sequences; the websites Peptide Ranker, GRAVY (Grand average of hydropathy), ProtParam, and Compute pI/Mw allow us easily to determine the possible physical and chemical properties of any peptide, such as pI (Isoelectric Point), Mw (Molecular weight) and potential active; use of molecular docking and quantum chemical calculation can help us to predict/or explain how a peptide works [2,4]. It is noted that in-silico peptide databases and software have been used to identify 10 novel bioactive DPP-IV, renin, and ACE inhibitory peptides from meat proteins [5], as well as tyrosinase inhibitory peptide FPY from walnut protein [6]. However, few reports have been presented to show the possibility of the in-silico tools in screening antioxidant peptides. 

Hazelnut, belonging to the family Betulaceae, is an important food source both for oil and protein supplies, in which edible oil occupies 60% of total dry weight, and protein is 15% [7]. Easy lipid oxidation is the main challenge for direct consumption of hazelnut due to the presence of about 90% unsaturated fatty acids in nuts [7,8]. However, as a major by-product of woody oil production, hazelnut protein is usually treated as feed for animals or fertilizers for soil due to its potential allergenicity to humans [9]. To date, limited data on characterizing the bioactive components of hazelnut protein have been reported [10], especially the hazelnut-derived peptides involved in the inhibition of lipid oxidation. Therefore, studies must be carried out to identify if it is possible to separate antioxidant peptides against oil oxidation from hazelnut protein according to the properties of amino acid residues by in-silico tools. 

In this study, a hybrid hazelnut (*Corylus. heterophylla* Fisch. × *Corylus. avellana* L., cv. Dawei) widely cultivated in the north part of China was selected to identify antioxidant peptides in terms of active amino acids (Met, Trp, and Tyr), Mw and GRAVY. The selection of protease was detected by searching expected cleavage sites from the database ExPASy ENZEMY. The peptides processed by the selected enzyme from hazelnut protein were sequenced, in which antioxidant peptides able to inhibit linoleic acid oxidation were identified by featuring the properties of active amino acid residues. Identified peptides were then used to protect hazelnut oil from oxidation in an oil-in-water emulsion system. Next, the geometries and active sites were visualized using quantum chemical computation to verify the significance of active amino acids in improving the antioxidant capacity of a biopeptide. The following are all results.

## 2. Materials and Methods

### 2.1. Materials

All reagents were of analytical grade. Peptides (22 dipeptides and 9 oligopeptides) were synthesized using a solid phase peptide synthesis method by Zhejiang Ontores Biotech Co., Ltd. (Hangzhou, China) with a purity of more than 90%. After cold-pressing for crude oil, defatted hazelnut flour (DHF) was obtained after a second solvent extraction to remove excess oil.

### 2.2. Experimental Analysis

#### 2.2.1. Inhibition Activity Assay of Linoleic Acid Oxidation

The ability of amino acids and peptides to inhibit lipid oxidation was determined using a linoleic acid emulsified model. Briefly, amino acids or peptides were dissolved in 50 mM phosphate buffer (pH = 7.0) and then added to a linoleic acid emulsion. In previous studies, different incubation temperatures were designed for linoleic acid oxidation assessment at 20 °C [11], 40 °C [4,12], and 60 °C [13], respectively. However, most studies selected 40 °C for linoleic acid oxidation assay. Subsequently, the reaction mixture was then incubated in tubes at 40 °C in dark for 48 h. The degree of linoleic acid oxidation was measured at 500 nm after mixing with 30% ammonium thiocyanate (dissolved in water) and ferrous chloride solution (dissolved in 3.5% HCl). Effect on the oxidation of linoleic acid was described as inhibition rate (IR), which was calculated by Equation (1). GSH was used as control and the results were expressed as GSH equivalents.

IR% = (1 − A_sample_/A_blank_) × 100 (1)

where A means absorbance value of samples (A_sample_) and blank (A_blank_) at 500 nm.

#### 2.2.2. Superoxide Radical Scavenging Activity Assay

The superoxide radical scavenging activity was measured at room temperature by monitoring the inhibition effect of pyrogallol auto-oxidation described by Li et al. [14]. Sample solutions (0.1 mL) were incubated at 25 °C for 10 min after added 2.8 mL Tris HCl-EDTA buffer (0.1 M, pH 8.0). The optical density was measured at 325 nm every 10 s for 240 s after mixing with pyrogallol solution (3 mM). The slopes represented rates of pyrogallol auto-oxidation. Superoxide radical scavenging activity was calculated by Equation (2). GSH was used as the control and the results were expressed as GSH equivalents.

K = (V_c_ − V_s_)/V_c_ × 100 (2)

where K means superoxide radical scavenging activity assay, %; V_c_ indicates auto-oxidation rate of pyrogallol; V_s_. stands for oxidation rate of pyrogallol after adding the sample.

#### 2.2.3. Metal Ion Chelation Activity Assay

Fe^2+^ chelation activities were determined according to the methods described by Zhang [15] with some modifications. First, 0.05 mL of 2 mM FeCl_2_ was mixed with 0.1 mL of 5 mM ferrozine. After the addition of samples solutions, the final volume was increased up to 3 mL with ultrapure water. The absorbance change was measured at 562 nm after being incubated for 10 min at room temperature. The metal ion chelation activity was calculated by Equation (3).

Metal ion chelation activity% = (1 − A_sample_/A_blank_) × 100 (3)

where A means absorbance value of samples (A_sample_) and blank (A_blank_) at 562 nm.

### 2.3. Synthesis of Dipeptides

A total of 22 dipeptides were synthesized according to amino acid activities and properties. The active amino acid residues located at the N-terminus are represented by the letter X; acidic amino acid (D, Asp), basic amino acid (H, His), and hydrophobic amino acid (P, Pro) residues located at the C-terminus were named the dipeptides of XD, XH, and XP, respectively. The dipeptides of XX consist of active amino acid residues located at both C-terminal and N-terminal. To evaluate the antioxidant capacity of all synthesized dipeptides containing active amino acids, four dipeptides without active amino acid, that is, IR with ID 8215, KP with ID 8218, AH and KD obtained from the BIOPEP database (http://www.uwm.edu.pl/biochemia/, accessed on 1 June 2020), were also artificially made the controls. 

### 2.4. BIOPEP Database Analysis

The database BIOPEP as a bioinformatics tool allows the detection of biologically active fragments within protein sequences. BIOPEP categorizes proteins as potential sources of bioactive fragments [16,17,18] and is used to characterize food-derived peptides [19]. According to the keyword “antioxidative”, 294 peptides were found, in which 86 biopeptides related to lipid oxidation were selected. Additionally, their molecular weight, GRAVY value (Calculated by gravy-calculator from http://www.gravy-calculator.de, calculated on 20 May 2020), and location of active amino acids were analyzed.

### 2.5. Selection of Protease

The cleavage specificity of an enzyme plays a determining role in proteolytic peptide release [20], so the proteolytic sites of the enzymes are accessible from ExPASy (https://enzyme.expasy.org/, accessed on 13 August 2020). According to the properties of antioxidant peptides collected from the BIOPEP database and dipeptides in our study, a desirable protease was selected depending on the cleavage specificity.

### 2.6. Preparation of Hazelnut Protein Hydrolysates

Hazelnut protein was isolated as Tatar et al. reported [21]. Briefly, DHF dissolved in distilled water was adjusted to a pH of 8.0 with 1% NaOH solution, and stirred for 30 min. Then, the supernatant was collected by centrifugation (9391× *g*, 20 min) and adjusted to a pH of 4.5 by 1% HCl solution for precipitation. Next, the precipitates were collected by centrifugation (3000× *g*, 20 min) and freeze-dried for powder harvest after being neutralized to pH 7.0 by a 0.2% NaOH solution.

Hazelnut protein hydrolysates were prepared according to the method described by Liu et al. with some modification [22]. Briefly, 1 g freeze-dried protein, after being mixed with 50 mL distilled water, was denatured at 90 °C for 15 min. Selected proteases were added to mixtures at room temperature. The enzyme amount, reaction time, optimal pH, and reaction temperature were depended on different proteases. For alkaline and neutral proteinases, these parameters are 10,000 U/g protein, 120 min, pH 8.5, 54 °C [22], and 17,000 U/g protein, 120 min, pH 7.0, 44 °C [23], respectively. Obtained hazelnut hydrolysates, after being heated at 100 °C for 10 min to inactivate enzymes, were readjusted to neutral pHs (1% HCl solution for hydrolysates of alkaline protease). Next, the hydrolysates, after being centrifuged at 3910× *g* for 15 min to remove a little sediment, were freeze-dried for peptide preparation. The lyophilized peptide powders were stored at −20 °C prior to use. The purity of hazelnut hydrolysates was measured by Folin-phenol protein quantitative assay [22].

### 2.7. Analysis of Peptide Sequence

First, 1 mg of the lyophilized hydrolysates powder, after mixed with 1 mL ultrapure water, was transferred to a 3 KD ultrafiltration tube. The mixture was centrifuged at 12,000× *g* at 4 °C for 10 min and repeated twice. Then the disulfide bond of hydrolysates was treated by reductive alkylation with 10 mM DTT (DL-Dithiothreitol) and 20 mM IAA (Iodoacetamide) before LC-MS/MS (Liquid chromatography-tandem mass spectrometry, Thermo Fisher Scientific, Waltham, MA USA) analysis. The peptides, after being desalted and freeze-dried, were resuspended in 2 to 20 μL of 0.1% formic acid for sequencing. The hazelnut peptides were sequenced by Beijing Bio-Tech Pack Technology Company Ltd., (Beijing, China).

### 2.8. DFT Calculations

DFT method (Density Functional Theory method) with B3LYP/6-311G(d,p) was used to optimize the geometries of the molecules. The optimized stable conformation was confirmed to be real minima by frequency calculation (no imaginary frequency). Additionally, HOMO, E-gap (expressed by E_LUMO-HOMO_ = E_LUMO_ − E_HOMO_) [24], and Fukui functions were applied to predict reactivity sites. Fukui functions (f^−^, f^+^, f^0^) were calculated using Equations (4)–(6) [25]. q from Equations (4)–(6) represents the atomic charge at the rth atomic site with the neutral (N), anionic (N + 1), and cationic (N-1) chemical species [26]. All calculations were performed using the Gaussian 09, Revision D.01 program package (Gaussian, Inc., Wallingford, CT, USA) [27].

f^+^ = q(N + 1) − q(N) for nucleophilic attack (4)

f^−^ = q(N) − q(N − 1) for electrophilic attack (5)

f^0^ = (q(N + 1) − q(N − 1))/2 for radical attack (6)

### 2.9. Inhibition of Hazelnut Oil Oxidation Assay

The oil-in-water emulsion was prepared according to a previous study. To obtain oil without any antioxidants, hazelnut crude oil was stripped by silica gel, activated charcoal, and sucrose [28]. The aqueous phase of the emulsion was prepared by dispersing 0.5 wt% Tween 20 in 10 mM phosphate buffer at pH 7.0 followed by stirring at room temperature for 20 min to ensure complete dispersion. Hazelnut oil-in-water emulsions were prepared by homogenizing 10 wt% oil phases with 90 wt% aqueous phases at ambient temperature using a high-speed blender for 2 min, followed by ultrasonic vibration for 20 min. Peptides or TBHQ (tert-Butylhydroquinone, as positive control) were added to this emulsion with a final concentration of 0.02% to test the inhibition ability of peptides against oil. Sodium azide (NaN_3_, 0.02% (*w*/*w*)) was used as an antibacterial agent.

Incubation temperature for the oil-in-water emulsion system was generally set at 37 °C [29] or 40 °C [30]. In this work, a longer incubation time was required for analyzing the possible oxidative products of oil-in-water emulsion compared to that of linoleic acid oxidation, so an incubation temperature of 37 °C was selected. During incubation at 37 °C for 14 days, the levels of lipid hydroperoxides as primary lipid oxidation products were monitored at regular intervals to assess the degree of oxidation of hazelnut oil. In brief, 0.3 mL of emulsions were breakdown by 1.5 mL of isooctane/2-propanol (3:1, *v*/*v*), vortexed, and then centrifuged at 1000× *g* for 2 min. Next, 200 μL of organic solvent phases were collected and mixed with 2.8 mL of methanol/1-butanol (2:1, *v*/*v*), followed by 15 μL ferrous iron solution (prepared by mixing 0.132 M BaCl2 and 0.144 M FeSO_4_, dissolved in 0.4 M HCl), and 15 μL ammonium thiocyanate solution (3.94 M, dissolved in water). The absorbance was measured at 510 nm after 20 min. Lipid hydroperoxides (μmol/g oil) were calculated using a standard curve prepared by cumene hydroperoxide (0, 20, 40, 80, 160, 300, and 400 μM) [31].

### 2.10. Statistical Analysis 

IBM SPSS 26.0 software (IBM Corporation. Armonk, NY, USA) was used for statistical analysis between groups. The data are expressed as the mean ± standard deviation (*n* = 3).

## 3. Results

### 3.1. Antioxidant Activity of Amino Acids and Dipeptides

**Figure 1:**
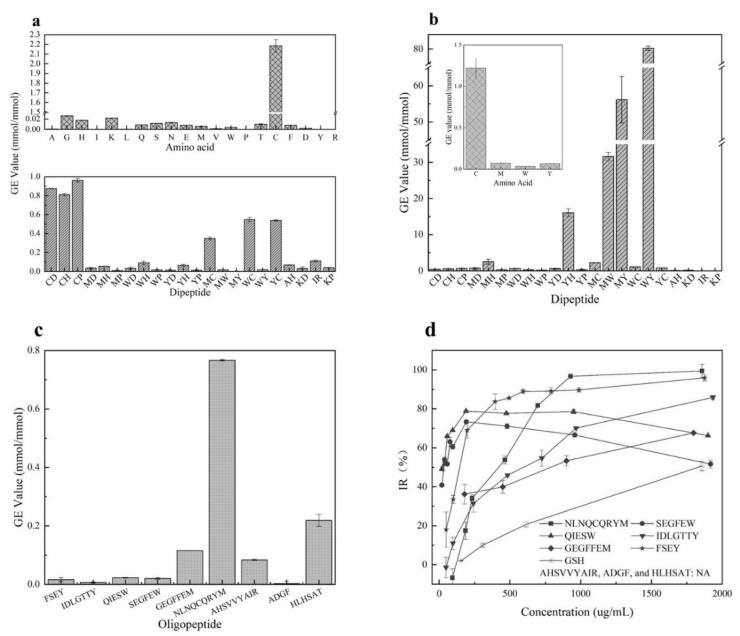
Antioxidant activities of amino acids and peptides. (**a**) means activity of amino acids and dipeptides against O_2_^•^^−^; (**b**) stands for activities of amino acids and dipeptides against linoleic acid; (**c**) shows effect of oligopeptides on the oxidation of O_2_^•−^; (**d**) indicates effect of oligopeptides on the oxidation of linoleic acid, AHSVVYAIR, ADGF, HLHSAT were not presented for the poor capacity (NA: No activity was observed at concentration less than 2000 μg/mL.).

It has been reported that GSH [32] with cysteine residue showed excellent O_2_^•−^ scavenging ability than peptides with other residues. Our study confirmed that dipeptides with cysteine residue showed stronger activities than others due to the strong ability of Cys in scavenging O_2_^•−^ (shown in Figure 1a). It was seen that the O_2_^•−^ scavenging activities of these synthesized dipeptides ranked in the order CP(>GSH) > CD > CH > WC > YC > MC > IR > WH > AH > YH > MH > KP > MD > WD > KD > WY > MW > WP > YP > YD > MP > MY. However, the dipeptides with W, M, or Y residues seem to be inactive in scavenging O_2_^•−^. Additionally, these synthesized dipeptides did not show any ability to chelate Fe^2+^ (data not shown).

Furthermore, as shown in Figure 1b, the amino acids Cys, Met, Trp, and Tyr were confirmed to have significant inhibition activity against linoleic acid oxidation after analyzing the antioxidant activities of 20 amino acids. The amino acid Cys showed stronger activity than GSH, followed by Tyr, Met, and Trp. The GE values of Cys, Met, Trp, and Tyr were 1.22 ± 0.12, 0.07 ± 0.01, 0.03 ± 0.01, and 0.07 ± 0.01 mmol/mmol, respectively. The remining amino acids did not show antioxidant activities. Moreover, 22 dipeptides were synthesized based on the inhibition activity of the 4 amino acids towards the oxidation of linoleic acid. It was observed that among these synthesized dipeptides, the peptide WY had the best antioxidant capacity with the GE value 80.29 ± 0.68 mmol/mmol, followed by peptides MY, MW, YH, MH, MC, WC(GSH), YC, MD, WD, CP, YD, CH, CD, YP, MP, WH, KD, WP and AH(0.12 ± 0.04 mmol/mmol) (IR and KP showed no inhibitory activity). Clearly, the more active amino acid residues existed, the stronger the inhibitory activity of dipeptides exhibited. Interestingly, Cys was observed to show an excellent antioxidation, but the dipeptides with Cys residue did not have more activity against linoleic acid oxidation than the dipeptides with Met, Trp, and Tyr residues. In addition, the dipeptides IR and KP did not show any activity in inhibiting linoleic acid oxidation even though they were reported to scavenge oxygen radicals [33]. Thus, data from our study indicated that the dipeptides containing Met, Trp, or Tyr residues should be selected as potential inhibitors of stopping linoleic acid oxidation.

### 3.2. BIOPEP Database Analysis

To evaluate the possible inhibition of antioxidants towards oil oxidation, bioactive peptides are collected from the BIOPEP database and various publications (they are listed in Table S1). Further analysis showed that the peptides whose molecular weight is from 200 to 800 plus a GRAVY value of −2 to 1 as the major proportion of these biopeptides had a possibility of stopping oil oxidation (as presented in Figure S1a,b). Interestingly, it is noted that almost three-quarters of the potential antioxidant peptides containing Tyr, Trp, Cys, or Met residues and mainly located at the C-terminus or N-terminus, especially Tyr residue (as presented in Figure S1c). Thus, it could be presumed that the peptides have a molecular weight of 200 to 800, GRAVY value of −2 to 1, and Tyr, Trp, Cys, or Met residues at N- or C- terminus should be selected as effective candidates for antioxidants.

### 3.3. Selection of Protease

To find a targeted peptide that consists of active amino acid residues at the N- or C- terminus, the database ExPASy ENZYME integrating available information about proteolytic sites and enzymes was used to select appropriate protease. The use of ExPASy ENZYME allows us easily to determine the cleavage site between all pairs of amino acids in the N- or C-terminal [34]. According to the preferential cleavage sites searched from ExPASy ENZYME (https://enzyme.expasy.org/enzyme-search-ec.html, accessed on 13 August 2020) and from a review [35], the alkaline proteinase (EC number: 3.4.21.62), chymotrypsin (EC number: 3.4.21.1) and pepsin A (EC number: 3.4.23.1) are likely to hydrolyze proteins to the peptides with Tyr, Trp, or Met as C-terminus or N-terminus (Seen in Table 1). Among the three proteases, alkaline hydrolysates exhibited the highest inhibition effect on linoleic acid oxidation [36]. To further reveal the preference of the proteases for producing Tyr, Trp, and Met residues, alkaline and neutral proteinases were chosen to “really” hydrolyze hazelnut protein. It was seen from Table 1 that alkaline proteinase hydrolysates exhibited a high inhibition rate of 95.11 ± 0.17% after 1 mL of the hydrolysates were added to linoleic acid emulsions incubated at 40 °C for 48 h. However, the inhibition rate of linoleic acid by neutral hydrolysates was 81.44 ± 1.94%. In addition, protein hydrolysates hydrolyzed by alkaline + neutral proteinase showed an in-between inhibition rate (83.35 ± 1.02%). Similar to the result reported by Ngamsuk [37], that alkaline proteinase was found to give high activity hydrolysate compared to neutrase and mix.

Furthermore, the purity of hazelnut hydrolysates processed by alkaline proteinase was 73.66 ± 2.50%. It was clear from our data that the proteinase alkaline could be appropriately selected for processing the antioxidant peptides from hazelnut protein. The alkaline protease hydrolysates were freeze-dried for further study.

### 3.4. Screening of Antioxidant Peptides

Peptides with molecular weights less than 3 kD, which produced from hazelnut protein were sequenced for the screening of potential antioxidants. Furthermore, the peptides which have Tyr, Trp, and Met residues, whose molecule weight is less than 800 and with GRAVY value of −2 to 1 are desirable for us, as sorted out in Table 2. It was seen that seven peptides from hazelnut protein, designed from No.1 to No.7, were screened as potential antioxidants due to up to the desired requirements. Next, they were artificially prepared as follows regarding the determined amino acid sequences. The five peptides FSEY, QIESW, SEGFEW, IDLGTTY, and GEGFFEM were artificially made based on the active amino acids of their C-terminal. The peptide AHSVVYAIR (designed as No.6) was synthesized in terms of Tyr-containing residue in its middle position. The peptide NLNQCQRYM (named as No.7) was synthesized because of the existence of Tyr, Cys, and Met residues. Besides this, two reported peptides HLHSAT and ADGF from hazelnut protein were artificially synthesized according to their ability to scavenge ABTS and DPPH radical [22,23]. Thus, the four peptides NLNQCQRYM, AHSVVYAIR, HLHSAT, and ADGF were artificially prepared as the control, and they were designed to clarify the feasibility of amino acid residues in screening antioxidant peptides. 

As predicated by us, six synthesized peptides showed a significant impact on retarding the oxidation of linoleic acid (Figure 1d). Peptides FSEY and NLNQCQRYM showed the best antioxidant activity, followed by QIESW, SEGFEW, IDLGTTY, and GEGFFEM. However, the inhibition of these synthesized peptides against linoleic acid oxidation functioned in a dose-dependent manner. Peptide NLNQCQRYM with a concentration higher than 900 µg/mL performed excellent activity in stopping linoleic acid from oxidation, while inhibition rates of the peptides QIESW and SEGFEW which contain Trp residue were less than 80% when their concentrations exceeded 200 µg/mL. It was noted that peptide AHSVVYAIR showed a poor capacity, and its IR% was only 50 even when its concentration was elevated to 5000 µg/mL (Figure 1 not shown). Our work indicated that peptides HLHSAT and ADGF, which have been reported to have ABTS and DPPH radical scavenging capacity [22,23] did not show any inhibition against the oxidation of linoleic acid. Moreover, the peptides that contain Cys residue showed perfect O_2_^•−^ scavenging activity compared to others, as these dipeptides did (see Figure 1c). Clearly, data from the artificially synthesized peptides support our assumption that the occurrence of active amino acid residues plays a crucial role in promoting the antioxidant capacity of a peptide. Featuring the properties of the amino acid residues should be a simple and feasible tool for the quick selection of desirable antioxidant peptides from hazelnut protein. Besides this, despite the excellent impact on retarding linoleic acid oxidation of FSEY and NLNQCQRYM, FSEY was selected for further study as the peptide falls within a molecular weight of 200 to 800 and GRAVY value of −2 to 1, as well as containing Tyr residue at the C-terminal.

### 3.5. DFT Calculation of Peptides 

#### 3.5.1. Frontier Molecular Orbital Energy of Peptides

To convince the antioxidant performance of four active amino acids, 22 synthesized dipeptides were prepared, and their quantum chemical parameters were obtained by DFT calculations [38]. As shown in Table 2, the frontier molecular orbital energy of each dipeptide, expressed by E_HOMO_ (Energy of highest occupied molecular orbital) and E_LUMO_ (Energy of lowest unoccupied molecular orbital), represents the active level of 22 synthetics [26]. Theoretically, a higher E_HOMO_ means more unstable electrons, which are more likely to scavenge free radicals as hydrogen donors. It was seen from Table 3 that the free radical-scavenging ability of these dipeptides, according to their calculated E_HOMO_, ranked from high to low in the order WH > WP > WY > WC > WD > MW > MH > YH > MY > YP > MP > CH > MD > MC > YD > IR > YC > KP > KD > AH > CP > CD. Moreover, a lower energy gap (E-gap) represents a higher chemical reactivity [39]. It means that the antioxidative activity of 22 dipeptides, based on their energy gap, ranked from strong to poor in the order WH > WC > WY > WP > WD > MW > MC > MH > MD > MY > YH > YC > YD > YP > CH > KD > CD > IR > AH > MP > KP > CP. To evaluate the real antioxidation of the 22 peptides, their inhibition towards linoleic acid oxidation was examined (see Figure 1b and Table 3). As a result, the best-synthesized dipeptide which retards the oxidation of linoleic acid was WY, followed by peptides MY, MW, YH, MH, MC, WC, YC, MD, WD, CP, YD, CH, CD, YP, MP, WH, KD, WP, AH, IR, and KP. It was noted that the dipeptides including KP, KD, IR, and AH with low E_HOMO_ and high E-gap values had a low antioxidant capacity, whereas the dipeptides like WC, WY, MW, MC, MY, MH, and YH which have high E_HOMO_ and low E-gap values showed a higher inhibition towards the oxidation of linoleic acid (see Table 3). Although several dipeptides had a poor activity in inhibiting the oxidation of linoleic acid, most of the synthesized dipeptides showed good antioxidant activity, especially these dipeptides containing W, M, or Y residue. It means that there is a corresponding relationship between the antioxidant activity of a dipeptide and its frontier molecular orbital energy.

**Figure 2:**
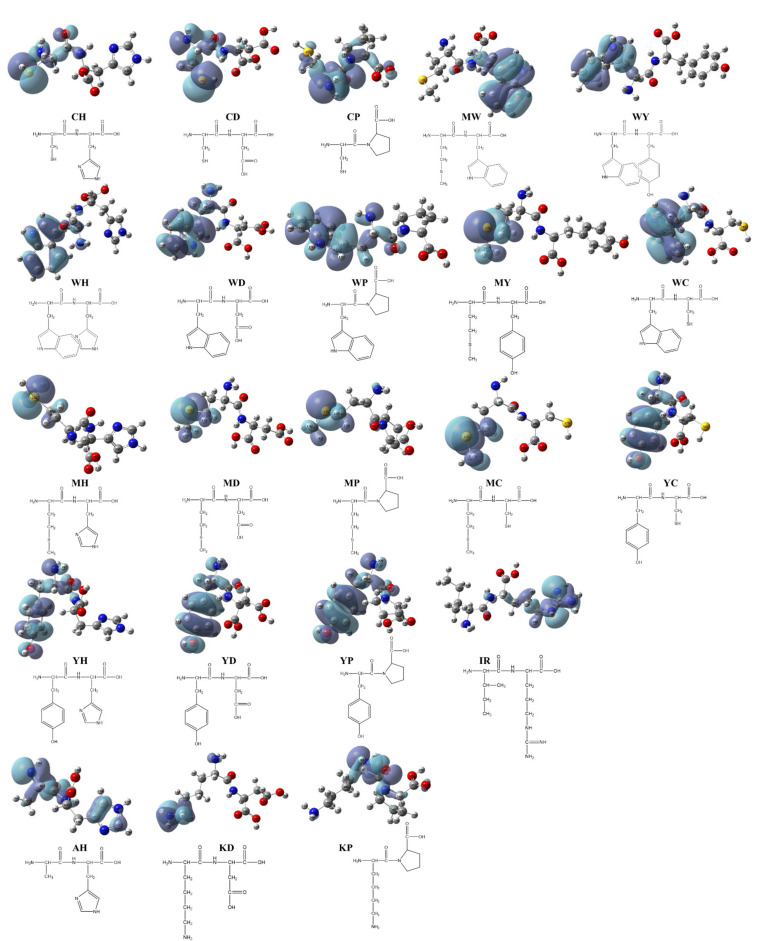
HOMO distribution of the dipeptides. Note: red ball represents the oxygen atom; blue ball represents the nitrogen atom; dark gray ball represents the carbon atom; yellow ball represents the sulfur atom; light gray ball represents the hydrogen atom.

**Figure 3:**
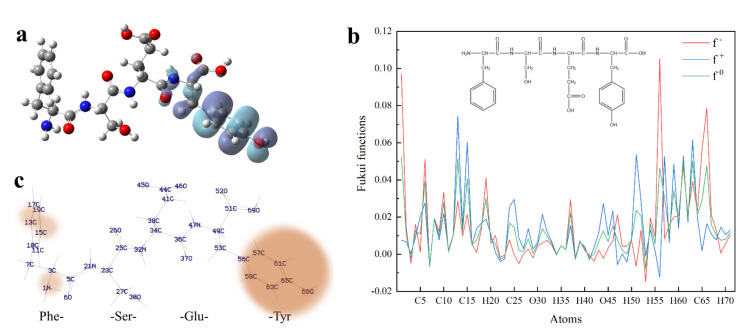
Quantum chemical parameters of FSEY. (**a**) shows HOMO distribution of peptide FSEY; (**b**) stands for Fukui functions; (**c**) means the predicting sites of FSEY more prone to a nucleophilic, electrophilic or radical attack. Note: red ball represents the oxygen atom; blue ball represents the nitrogen atom; dark gray ball represents the carbon atom; light gray ball represents the hydrogen atom.

To clarify the possible active site of each peptide, we constructed the dimensional structures of 22 synthesized dipeptides as well as FSEY. It is well known that HOMO always acts as the active site of any organic compound [40]. As shown in Figure 2 and Figure 3a, the HOMOs of all dipeptides as well as FSEY are located at their active amino acid residues, that is, C, W, M, or Y. It indicates that these amino acids will firstly lose their electrons once when interacting with free radicals [41]. Clearly, data from the analysis of the HOMO site and linoleic acid oxidation inhibition confirmed the significance of active amino acid residues in determining the activity of antioxidant peptides. The results from Hougland also et al. supported our findings [42].

#### 3.5.2. Fukui Function

To predict which atom would be most susceptible to a nucleophilic or electrophilic attack, peptide FSEY was selected to investigate the tendency by Fukui functions. Fukui function is a local reactivity parameter, which is widely used for molecular reactivity analysis, indicating the tendency of a molecule to lose or gain an electron thus predicting which atom in the molecule would be more prone to a nucleophilic or electrophilic attack. When a molecule prefers to accept an electron, the Fukui function is f^+^, it is the index of nucleophilic attack. While when a molecule has a tendency to lose an electron, the Fukui function is f^−^ and is also termed as the index of electrophilic attack [22]. In our study, the individual atomic charges were calculated by natural population analysis (NPA) with B3LYP/6-311G (d, p) basis set. For all atomic sites of peptide FSEY, their Fukui functions (f^−^, f^+^, f^0^) were presented in Figure 3b,c, respectively.

Blue, red, and green colors in Figure 3b represent nucleophilic, electrophilic, and radical attacks, respectively. It is found that nucleophilic, electrophilic, and radical attacks of peptide FSEY are located at N_1_, C_13_, C_15_, C_51_, C_56_, C_57_, C_59_, C_61_, C_63_, C_65_, and O_66_ atoms, especially at the atoms of Tyr residue. These results further highlighted a fact that Tyr residue should act as a biological activity site, as shown in Figure 3c. Moreover, the molecular reactivity site of peptide FSEY, as indicated by Fukui functions, totally corresponded to that of its HOMO.

### 3.6. Effects of Tyrosine Residue’s Location on the Antioxidant Activity of Peptides

**Figure 4:**
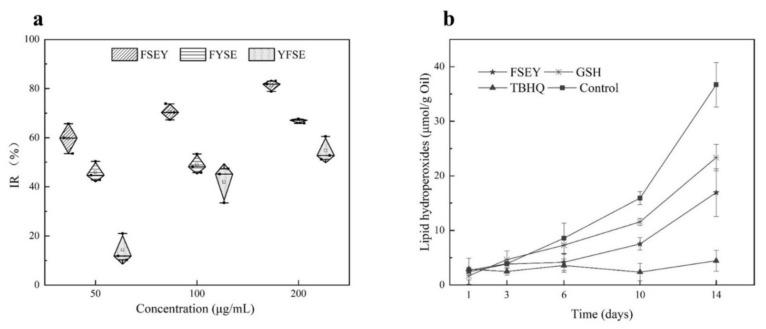
Antioxidant activities of peptides. (**a**) stands for activities of FSEY, FYSE, and YFSE on the oxidation of linoleic acid; (**b**) represents the effects of the antioxidants on oil oxidation, GSH and TBHQ as positive controls.

To clarify how the position of tyrosine residue governs the antioxidant activity of a peptide against free radicals, three peptides FSEY, FYSE, and YFSE were selected to examine their inhibitions towards the oxidation of linoleic acid (see Figure 4a). The three peptides have the same composition of amino acids but have different locations for tyrosine residue. After incubated with linoleic acid at 40 °C for 48 h, the peptide FSEY showed the strongest ability in stopping the oxidation of the fat acid compared to others. The peptide YFSE had the lowest antioxidant activity. It is concluded that if tyrosine residue is located at the C-terminal, the Tyr-containing peptide should have a stronger activity against linoleic acid radical, as shown in Figure 4a.

### 3.7. Application of Hazelnut Peptide FSEY in Inhibiting Lipid Oxidation

To validate the antioxidant ability of selected peptides in a real-emulsion, 0.02% hazelnut-original peptide FSEY was added to a hazelnut oil-in-water emulsion system for the evaluation of antioxidant activity against oil rancidity. It was seen that the hazelnut-derived peptide FSEY inhibited the rancidity of oil very well by analyzing hydroperoxides on days 1, 3, 6, 10, and 14 (see Figure 4b). Furthermore, the antioxidant activity of peptide FSEY was compared with that of TBHQ, which is a commercial additive for protecting the oil from rancidity, as well as GSH, which is an antioxidant peptide (see Figure 4b). After incubation at 37 °C for 14 days, hydroperoxides of the emulsion system was 36.69 µmoL/g oil without antioxidant, whereas that was 16.94 µmoL/g oil, 22.35 µmoL/g oil, and 4.44 µmoL/g oil in the presence of FSEY, GSH, and TBHQ, respectively. It was clear that peptide FSEY showed a higher ability than GSH in controlling lipid oxidation, but lower activity than TBHQ. These results indicated that hazelnut-original peptide FSEY could be used as an antioxidant in the emulsion system for delaying the rancidity of oil. In addition, due to a weak ability in O_2_^•−^ scavenging and Fe^2+^ chelation, we speculate that the peptide FSEY act as a radical scavenger by contributing phenolic hydrogen atom to peroxyl radical.

## 4. Discussion

**Figure 5:**
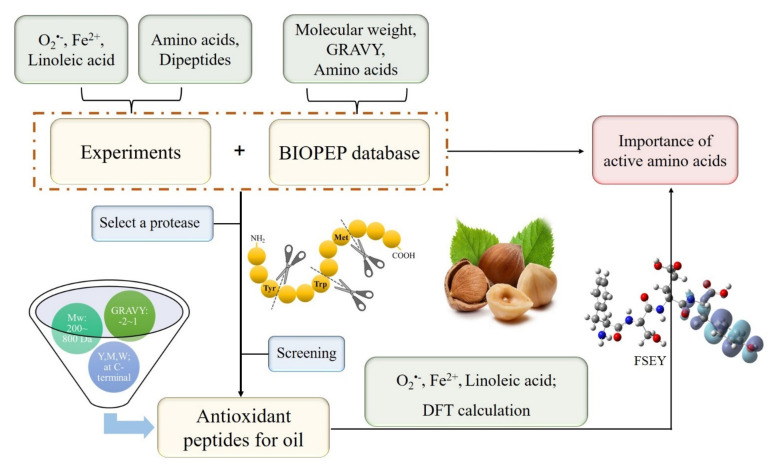
Screening antioxidant peptides depending on properties of amino acid residues.

Generally, antioxidant peptides against oil oxidation should act as one or more roles, that is, containing hydrophobic amino acids which expose more active sites to terminate lipid chain reaction; having free radical scavenging agents (such as O_2_^•−^ and peroxyl radical) or as metal ions chelating agents; and possessing strong lipase-inhibitory activities [2]. Obviously, the efficiency of antioxidation peptides in an emulsion system depends greatly on their ability to present more active sites, scavenge superoxide radicals or peroxyl radicals, and chelate metal ions. In addition, GSH was selected as a positive control for the outstanding antioxidant properties. Our study was pictured in Figure 5. As is shown, we used chemical experiments and physical properties of biopeptides as well as DFT calculations to verify which properties of the amino acid residues could be used to screen antioxidant peptides.

### 4.1. Chemical Test: We Found Four Key Amino Acid Residues

The radicals scavenging capacity of a peptide depends greatly upon its amino acid residues, especially upon Tyr, Trp, Met, and Cys. To illustrate this point, various tests firstly were carried out to confirm that Tyr, Trp, and Met and dipeptides containing these residues have excellent antioxidant capacities against the oxidation of linolic acid. It was observed that the absence of these residues caused the dipeptides to lose their activities (see Figure 1b). These amino acids, which are crucial in scavenging free radicals, have been reported by several research works. Amino acids Tyr, Trp, Cys, and Met as well as peptides containing these amino acids showed activities against ABTS radicals and oxygen radicals (ORAC, oxygen radical absorbance capacity) [43], active against ROS or RNS [3], and effectively against AAPH-induced peroxyl radicals [41].

### 4.2. DFT Calculation: Tyr, Trp, Met, and Cys Are Active Sites

Theoretically, quantum chemical computations can gain prediction of behaviors of organic compounds, such as their structural features and chemical reactivity, and therefore, help to analyze the relationship between the biological potencies and the type of compounds [38]. The distributions of HOMO correspond to the active sites of the peptides able to scavenge free radicals [26]. By our DFT calculations, HOMOs of the tested peptides are located at their active amino acids, that is, Cys, Trp, Met, or Tyr (see Figure 2). The HOMOs of some peptides including EAAY, PMRGGGYHY, PMRGGYHY, PMRGYHY, PMRYHY, and YHY have been reported to be concentrated on the phenolic hydroxyl structure in Tyr [41]. The peptides PVETVR, QEPLLR, RDPEER, and LDDDGRL have the HOMOs of guanidyl in Arg, and the active sites of peptides KELEEK, DAAGRLQE, and GFAGDDAPRA are located at Lys-Glu, Gly, and Asp [24,39]. Clearly, data from HOMOs addressed that the residues Cys, Trp, Met, or Tyr are key components responsible for the antioxidant activity of the tested peptides in our study.

Generally, a high E_HOMO_ or a low E-gap value means flexible chemical reactivity and could be used to predict the antioxidant activity of each peptide [24]. As predicted in our study, seven synthetic dipeptides, having a higher E_HOMO_ and a lower E-gap value, showed a good ability to inhibit the oxidation of linoleic acid. It is found the seven antioxidant dipeptides possess the active residues Tyr, Trp, or Met. The presence of Tyr, Trp, and Met significantly enhanced the antioxidant activity of these dipeptides compared to other tested peptides (see Table 3). In a similar study, Wang et al. used E_HOMO_ and E-gap to predicate the antioxidant activity of five peptides with only one exception [39]. Experiments conducted by Wu et al. also indicated that E_HOMO_ and E-gap were feasible to describe the antioxidant behaviors of a set of man-made peptides, which were designed from the parent peptide “PMRGGGGYHY” [41]. Consistent with other studies reported [44], the presence of active residues Tyr, Trp, or Met as well as high E_HOMO_ and low E-gap should be the characteristics of a peptide responsible for inhibiting the oxidation of linoleic acid. Amino acids, Tyr and Trp, act as active sites were also confirmed by Molecular docking. Wang et al. found that Trp1 and Tyr4 in peptide WLSYPMNPATGH could form hydrogen bonds with DPPH, which means responsible of Trp and Tyr in scavenging DPPH free radical. These two emerging approaches are helpful in analyzing antioxidative products, meanwhile, DFT calculation is a useful tool in screening antioxidant peptides [45].

### 4.3. BIOPEP Database Analysis: Rules of Molecular Weight, GRAVY Value and Active Amino Acid Residue’s Location

Few reports have focused on how a molecular weight, GRAVY value, and position of amino acid residues affect the capacity of a peptide against oil oxidation yet. In our study, a peptide in the BIOEPE database falling within a molecular weight of 200 to 800, GRAVY value of −2 to 1, and active amino acid residues at N- or C- terminus produced a strong inhibition towards lipid oxidation, as shown in Table S1 and Figure S1.

Regarding molecular weight or numbers of amino acid residues, numerous studies have indicated that peptides containing amino acid residues between 2 and 11 [46] or weighing less than 1000 Da [47] will exhibit good antioxidant ability. Peptides with more molecular weights (>2000 Da) easily decrease in their antioxidant activity due to the hiding of the active site [47]. It is well-known that the interfacial phase, the contact region between the oil phase and the aqueous phase, is the critical region in the system with regard to the development of lipid peroxidation [48]. Thus, in a given emulsion system, GRAVY value could not be ignored for antioxidant estimation, because a higher GRAVY means higher hydrophobicity. Various researches have proposed that peptides with higher hydrophobicity can protect linoleic acid from oxidation by donating protons to hydrophobic peroxy-radicals [49,50]. In our study, it is seen that peptides from BIOPEP database which able to stop lipid oxidation have GRAVY values ranging from −2 to 1 (see Table S1 and Figure S1b). Thus, a GRAVY value of −2 to 1 was proposed to be an ideal criterion for looking for antioxidant peptides from protein hydrolysate. A recent study has overviewed the roles of amino acid composition and sequence in conferring the antioxidant activities of peptides. The phenolic hydroxyl of Tyr, the indolyl of Trp, the thiol group of Cys, and the thioether of Met are regarded to act as hydrogen donors for free radicals [2,39,51]. Our study has found that the most of antioxidative peptides which are searched from the database BIOPEPE have the residues Tyr, Trp, Cys, or Met. Similarly, the antioxidant activities of peptides LGFEY and LGFYY were attributed to the presence of Tyr residues [52]. Concerning the inhibition of lipid oxidation, most peptides searched from the BIOPEPE database are observed to have active amino acid residues located at C- terminus. In our case, a linoleic acid oxidation system was designed to confirm the strong antioxidant activity of the peptide FSEY having Tyr residue located at the C-terminal (see Figure 4a). HOMO analysis from the synthesized peptides FYSE and YFSE also presents evidence that the reaction sites are all located at Tyr (see Figure S2). Similarly, studies done by Guo et al. [53] and Torkova et al. [54] indicated that peptides having Tyr residue located at the C-terminus strongly scavenged hydroxyl-radical, hydrogen-peroxide, and peroxyl radicals. While these peptides exhibited a better inhibition against ABTS cation radical when Tyr residue is located at the N-terminus [54]. Clearly, the types and positions of amino acid residues should be considered in searching for an antioxidant peptide from protein.

In conclusion, a wanted antioxidant peptide could be quickly screened by determining the types and location of amino acid residues as well as molecular weight and GRAVY, especially Tyr, Trp, Cys, and Met which act as H donors. Among the four amino acids, Tyr-containing peptides show a prominent antioxidant activity, especially when it is located at the C-terminus.

## 5. Conclusions

In our study, amino acids, Met, Tyr, Try, and peptides containing these active amino acids show antioxidant activity against linoleic acid radicals. These amino acid residues, as Tyr residue in peptide FSEY does, act as an active site for scavenging lipid free radicals. More meaningfully, the active amino acid residues located at C-terminal are more active than other positions. Compared to traditional technology for manufacturing bioactive peptides, our work presents a practical route able to successfully screen desirable high-activity antioxidant peptides from hazelnut protein hydrolysates by featuring the properties of amino acid residues. Our technical route consists of two steps. Firstly, peptides from hazelnut protein hydrolyzed by alkaline protease are sequenced; secondly, the peptides falling within a molecular weight of 200 to 800 and GRAVY value of −2 to 1 as well as containing Tyr, Met, Trp residues at C- terminus are selected for antioxidant candidates inhibiting the oxidation of the oil. Using this route successfully releases a peptide from hazelnut protein which inhibits oil oxidation very well. To our knowledge, it is the first attempt to prepare antioxidant peptides based on the properties of amino acid residues. Perhaps, the new findings out of our work will be beneficial for screening bioactive peptides from various protein resources with different purposes.

## Figures and Tables

**Table 1:** Preferential cleavage sites of several proteinases and inhibition rate of hazelnut protein hydrolysates against oxidation of linoleic acid.

EC Number	Name	Preferential Cleavage Sites	Inhibition Rate (IR, %)
3.4.21.1	Chymotrypsin	Cleaves, Tyr-|-Xaa, Trp-|-Xaa, Phe-|-Xaa, and Leu-|-Xaa	—^1^
3.4.21.62	Alcalase Novo/ *Bacillus subtilis* alkaline proteinase	Hydrolysis of proteins with broad specificity for peptide bonds, especially aromatic or hydrophobic amino acids. Cleaves, Glu-|-Xaa, Met-|-Xaa, Leu-|-Xaa, Tyr-|-Xaa, Lys-|-Xaa, Trp-|-Xaa, and Gln-|-Xaa	95.11 ± 0.71
3.4.23.1	Pepsin A (pH = 1.3)	Preferential cleavage, hydrophobic, preferably aromatic. Cleaves, Phe-|-Val, Gln-|-His, Glu-|-Ala, Ala-|-Leu, Leu-|-Tyr, Tyr-|-Leu, Gly-|-Phe, Phe-|-Phe, and Phe-|-Tyr	—^1^
3.4.24.27	Bacillolysin/ *Bacillus subtilis* neutral proteinase	Cleaves, Xaa-|-Leu> Xaa-|-Phe	81.44 ± 1.94

Note: 1 means not analyzed.

**Table 2:** Sequences of synthetic peptides containing Tyr, Trp, Cys, and Met residues.

**Number**	**Sequence**	**Length**	**Mw (Da)**	**GRAVY**	**Features**
1	FSEY	4	544.55	−0.70	With Tyr, Trp, or Met residue at the C-terminal.
2	QIESW	5	661.70	−0.84	
3	SEGFEW	6	753.75	−1.05	
4	IDLGTTY	7	781.85	0.24	
5	GEGFFEM	7	815.88	−0.04	
6	AHSVVYAIR	9	1015.16	0.74	With Tyr residue in the sequence.
7	NLNQCQRYM	9	1169.33	−1.29	With Cys, Tyr, and Met residues in the sequence.
8	HLHSAT	6	664.71	−0.38	With ABTS and DPPH radical scavenging ability.
9	ADGF	4	408.40	0.18	

Notes: A = Alanine, R = Arginine, N = Asparagine, D = Aspartic Acid, C = Cysteine, E = Glutamic Acid, Q = Glutamine, G = Glycine, H = Histidine, I = Isoleucine, L = Leucine, K = Lysine, M = Methionine, F = Phenylalanine, P = Proline, S = Serine, T = Threonine, W = Tryptophan, Y = Tyrosine, V = Valine. HLHSAT and ADGF are antioxidant peptides obtained from hazelnut in other studies [22,23].

**Table 3:** Frontier molecular orbital energy(eV) and GE (mM/mM) values of the dipeptides against the oxidation of linoleic acid.

	Dipeptide	E_HOMO_	E_LUMO_	E-Gap	GE Value
With GE value higher than 1	WC	−5.61	−0.73	4.87	1.13 ± 0.04
	WY	−5.59	−0.61	4.98	80.28 ± 0.68
	MW	−5.91	−0.75	5.15	31.59 ± 1.13
	MC	−6.21	−0.88	5.33	2.24 ± 0.03
	MY	−6.08	−0.59	5.49	56.17 ± 6.44
	MH	−5.91	−0.52	5.39	2.56 ± 0.68
	YH	−5.99	−0.34	5.65	16.06 ± 1.09
With GE value between 0.5~1	WD	−5.75	−0.61	5.13	0.70 ± 0.02
	MD	−6.17	−0.73	5.45	0.75 ± 0.21
	YC	−6.29	−0.63	5.66	0.85 ± 0.04
	YD	−6.26	−0.60	5.66	0.64 ± 0.08
	CH	−6.17	−0.47	5.70	0.57 ± 0.04
With GE value less than 0.5	WH	−4.77	−0.29	4.48	0.33 ± 0.03
	WP	−5.59	−0.52	5.07	0.20 ± 0.00
	YP	−6.14	−0.47	5.67	0.36 ± 0.12
	KD	−6.42	−0.68	5.75	0.22 ± 0.03
	CD	−6.63	−0.76	5.88	0.46 ± 0.01
	IR	−6.27	−0.16	5.94	0.00 ± 0.00
	AH	−6.54	−0.34	6.00	0.12 ± 0.04
	MP	−6.16	−0.14	6.02	0.35 ± 0.04
	KP	−6.34	−0.05	6.29	0.00 ± 0.00
	CP	−6.57	−0.18	6.39	0.70 ± 0.05

## Data Availability

The data is contained within the article or supplementary material.

## Supplementary Materials

The following are available online at https://www.mdpi.com/article/10.3390/antiox11010127/s1, Supplementary Materials to this article contains Table S1. Biopeptides collected from BIOPEP database and other publications, Figure S1. Analysis of the biopeptides harvested from BIOPEP database and other publications, Figure S2. The highest occupied molecular orbital (HOMO) of FYSE and YFSE.
