# Supplementary material for: A Novel Insight into Screening for Antioxidant Peptides from Hazelnut Protein: Based on the Properties of Amino Acid Residues
Source: Antioxidants (Basel). 2022 Jan 6;11(1):127. doi: 10.3390/antiox11010127 (PMC8772696; doi:10.3390/antiox11010127)
Supplement: Supplementary file 1 [file antioxidants-11-00127-s001.zip › antioxidants-1504938-supplementary.pdf]

Supplementary data:

Table S1. Bio-peptides collected from BIOPEP database and other publications

ID no.	Peptide sequence	GRAVY	Mw	Ref.
**Oxidation of linoleic acid**				
3296	HHP	-2.67	389.40	[1]
3297	YHH	-2.57	455.45	
3298	HHPL	-1.05	502.56	
3299	LHPH	-1.05	502.56	
3300	PHH	-2.67	389.40	
3301	HLH	-0.87	405.44	
3302	LHH	-0.87	405.44	
3303	HPLH	-1.05	502.56	
3304	LLPHHH	-0.60	752.86	
3305	LH	0.30	268.30	
3306	HPHL	-1.05	502.56	
3307	PYY	-1.40	441.46	
3308	HHLP	-1.05	502.56	
3309	LPYY	-0.10	554.62	
3310	LYPY	-0.10	554.62	
3311	HPH	-2.67	389.40	
3312	LLHH	0.30	518.60	
3313	PLHH	-1.05	502.56	
3314	LLPH	0.70	478.58	
3315	HLHP	-1.05	502.56	
3316	HLPH	-1.05	502.56	
3317	HL	0.30	268.30	
3318	LPHH	-1.05	502.56	
3319	HH	-3.20	292.28	
3320	HHPLL	-0.08	615.72	
3809	LQSGDALRVPSGTTYY	-0.37	1727.8	[2]
3810	MQFHT	-0.54	662.75	
3811	PHCKRM	-1.47	787.82	
3826	LVNPHDHQN	-1.56	1073.1	
7867	GYY	-1.00	401.39	[3]
7945	YYY	-1.30	507.51	[4]
7946	YYG	-1.00	401.39	
7967	YGY	-1.00	401.39	
8253	LEELEEELEGCE	-0.92	1438.30	[5]
8282	ISELGW	0.45	703.78	[6]
8433	FLKPLFNAALKLLP	0.98	1585.00	[7]
8439	YLMSR	-0.18	668.80	[8]
8440	VLYEE	-0.06	651.70	
8441	MILMR	1.52	662.90	

8950	WCTSVS	0.58	698.61	[9]
8955	PYSFK	-0.96	640.72	[10]
8956	GFGPGL	0.63	546.61	
8957	VGGRP	-0.54	484.54	
9370	VKRRGQDCIHGFCSD	-0.78	1754.66	[11]
9371	GQFNDKRWIPFG	-1.01	1464.63	
9372	APIRMWYMYRKLTDMEPKPVA	-0.52	2597.14	
9450	LTEQESGVPVMK	-0.32	1317.52	[12]
**Thiobarbituric acid reactive substances**				
8463	VPKNYFHDIV	-0.13	1231.40	[13]
8464	LVMFLDNQHRVIRH	-0.05	1778.11	
8465	FVNQPYLLYSVHMK	0.11	1739.05	
9130	VAWRNRCKGTD	-1.22	1322.33	[14]
9131	WRNRCKGTD	-2.16	1152.11	
9132	AWIRGCRL	0.29	991.04	
9133	WIRGCRL	0.07	919.96	
9134	IRGCRL	0.23	733.75	
**Oxidation in liposomes**				
8480	YPELF	0.04	667.74	[15]
**β-carotene - linoleate bleaching (BCB)**				
9381	LTTLDSE	-0.23	777.82	[16]
9382	VVGGDGDV	0.55	716.73	
**Oxidation of the liver**				
945	CERPTCCEHS	-1.03	1214.86	[17]
**Oxidation of linoleic acid**				
	AVPYPQR	-0.93	829.94	[18]
	WPP	-1.37	398.46	[19]
	LW	1.45	317.38	[20]
	FD	-0.35	280.27	
	WL	1.45	317.38	
	WV	1.65	303.36	
	YLGAK	0.00	550.65	[21]
	GFR	-0.70	378.43	[22]
	GPCSR	-0.96	518.59	
	CFCTKPC	0.59	801.01	
	MCESASSK	-0.45	841.94	
	RGY	-2.07	394.43	[23]
	WIR	-0.30	473.58	
	VAW	1.70	374.44	

YGS	-0.83	325.32	[24]
WYGPD	-1.54	636.66	[25]
KLSDW	-1.06	647.72	
WEGPK	-2.06	615.69	[26]
LTEQESGVPVMK	-0.32	1317.51	[12]
YP	-1.45	278.30	[27]
YPPAK	-1.32	574.67	[28]
LNLPTAVYMVT	1.08	1221.46	[29]
HLFGPPGKKDPV	-0.78	1291.50	[30]
KHNRGDEF	-2.46	1002.05	[31]
NADFGLNGLEGLA	0.20	1290.37	[32]
NGLEGLK	-0.59	729.82	
LPHSGY	-0.58	672.73	[33]

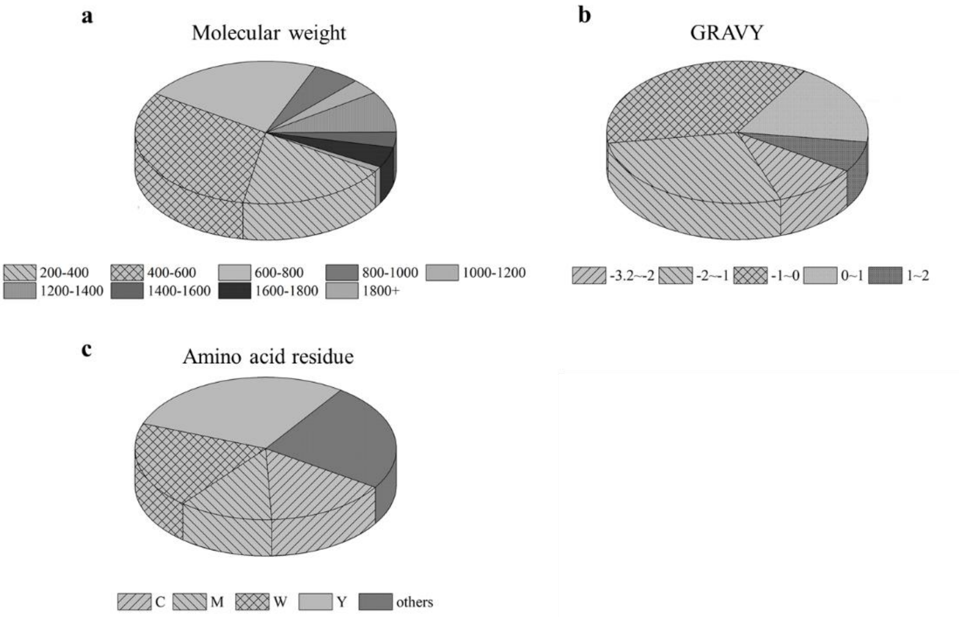

Figure S1. Analysis of the bio-peptides harvested from BIOPEP database and other publications

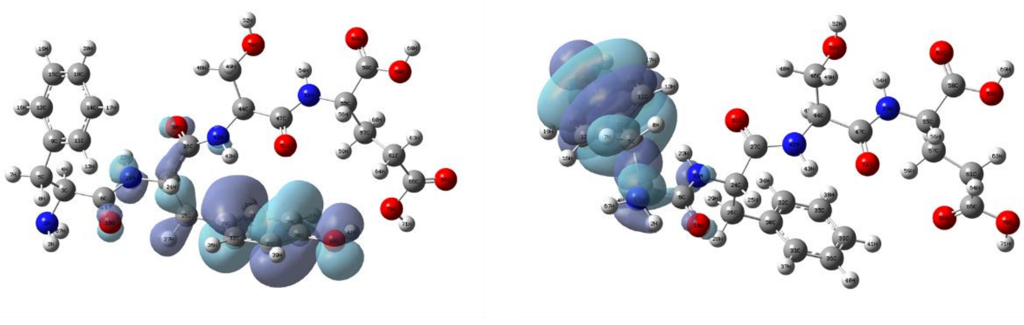

FYSE YFSE

Figure S2. The highest occupied molecular orbital (HOMO) of FYSE and YFSE

Reference

1. Chen, H.; Muramoto, K.; Yamauchi, F.; Nokihara, K. Antioxidant Activity of Designed Peptides Based on the Antioxidative Peptide Isolated from Digests of a Soybean Protein. *J. Agric. Food. Chem.* **1996**, *44*, 2619-2623.
2. Pihlanto, A. Antioxidative peptides derived from milk proteins. *Int. Dairy J.* **2006**, *16*, 1306-1314.
3. Yokomizo, A.; Takenaka, Y.; Takenaka, T. Antioxidative Activity of Peptides Prepared from Okara Protein. *Food Sci. Technol. Res.* **2002**, *8*, 357-359.
4. Saito, K.; Jin, D.H.; Ogawa, T.; Muramoto, K.; Hatakeyama, E.; Yasuhara, T.; Nokihara, K. Antioxidative Properties of Tripeptide Libraries Prepared by the Combinatorial Chemistry. *J. Agric. Food. Chem.* **2003**, *51*, 3668-3674.
5. Qian, Z.J.; Jung, W.K.; Kim, S.K. Free radical scavenging activity of a novel antioxidative peptide purified from hydrolysate of bullfrog skin, Rana catesbeiana Shaw. *Bioresour. Technol.* **2008**, *99*, 1690-1698.
6. Tsopmo, A.; Romanowski, A.; Banda, L.; Lavoie, J.C.; Jenssen, H.; Friel, J.K. Novel anti-oxidative peptides from enzymatic digestion of human milk. *Food Chem.* **2011**, *126*, p.1138-1143.
7. Mojallal-Tabatabaei, Z.; Asoodeh, A.; Asadi, F.; Nezafati, H.R. ACE-Inhibitory and Antioxidant Activity of Temporin-Ra Peptide: Biochemical Characterization and Molecular Modeling Study. 2014; pp. 493-500.
8. Chi, C.; Hu, F.; Wang, B.; Ren, X.; Deng, S.; Wu, C. Purification and characterization of three antioxidant peptides from protein hydrolyzate of croceine croaker (Pseudosciaena crocea) muscle. *Food Chem.* **2015**, *168*, 662-667.
9. Sudhakar, S.; Nazeer, R.A. Structural characterization of an Indian squid antioxidant peptide and its protective effect against cellular reactive oxygen species. *J. Funct. Foods* **2015**, *14*, 502-512.
10. Cai, L.; Wu, X.; Zhang, Y.; Li, X.; Ma, S. Purification and characterization of three antioxidant peptides from protein hydrolysate of grass carp (Ctenopharyngodon idella) skin. *J. Funct. Foods* **2015**.
11. Yu, H.; Qiao, X.; Gao, J.; Wang, C.; Cai, S.; Feng, L.; Wang, H.; Wang, Y.p. Identification and Characterization of Novel Antioxidant Peptides Involved in Redox Homeostasis of Frog, Limnonectes fragilis. *Protein Pept Lett.* **2015**, *22*, 776-784.
12. Hamid; Tanzadehpanah; Ahmad; Asoodeh; Jamshidkhan; Chamani. An antioxidant peptide derived from Ostrich (Struthio camelus) egg white protein hydrolysates. *Food Res. Int.* **2012**.
13. Najafian, L.; Babji, A.S. Isolation, purification and identification of three novel antioxidative peptides from patin (Pangasius sutchi) myofibrillar protein hydrolysates. *LWT - Food Sci. Technol.* **2015**, *60*, 452-461.

14. Carrillo, W.; Gomezruiz, J.A.; Miralles, B.; Ramos, M.; Barrio, D.A.; Recio, I. Identification of antioxidant peptides of hen egg-white lysozyme and evaluation of inhibition of lipid peroxidation and cytotoxicity in the Zebrafish model. *Eur. Food Res. Technol.* **2016**, *242*, 1777-1785.
15. Gobba, C.D.; Tompa, G.; Otte, J. Bioactive peptides from caseins released by cold active proteolytic enzymes from Arsukibacterium ikkense. *Food Chem.* **2014**, *165*, 205-215.
16. Chang, S.K.; Ismail, A.; Yanagita, T.; Mohd Esa, N.; Baharuldin, M.T.H. Antioxidant peptides purified and identified from the oil palm (Elaeis guineensis Jacq.) kernel protein hydrolysate. *J. Funct. Foods* **2015**, *14*, 63-75.
17. Zeng, W.C.; Zhang, W.H.; He, Q.; Shi, B. Purification and characterization of a novel antioxidant peptide from bovine hair hydrolysates. *Process Biochem.* **2015**, *50*, 948-954.
18. Rival, S.G.; Boeriu, C.G.; Wichers, H.J. Caseins and Casein Hydrolysates. 2. Antioxidative Properties and Relevance to Lipoxygenase Inhibition. *J. Agric. Food. Chem.* **2001**, *49*, 295-302.
19. Chi, C.; Hu, F.; Wang, B.; Li, T.; Ding, G. Antioxidant and anticancer peptides from the protein hydrolysate of blood clam (Tegillarca granosa) muscle. *J. Funct. Foods* **2015**, *15*, 301-313.
20. Guo, H.; Kouzuma, Y.; Yonekura, M. Structures and properties of antioxidative peptides derived from royal jelly protein. *Food Chem.* **2009**, *113*, 238-245.
21. Chen, C.; Chi, Y.J.; Zhao, M.Y.; Lv, L. Purification and identification of antioxidant peptides from egg white protein hydrolysate. *Amino Acids* **2012**, *43*, 457-466.
22. Huang, G.; Deng, J.; Chen, H.; Huang, S.; Liao, J.; Hou, W.; Lin, Y. Defensin protein from sweet potato (Ipomoea batatas [L.] Lam 'Tainong 57') storage roots exhibits antioxidant activities in vitro and ex vivo. *Food Chem.* **2012**, *135*, 861-867.
23. Rao, S.; Sun, J.; Liu, Y.; Zeng, H.; Su, Y.; Yang, Y. ACE inhibitory peptides and antioxidant peptides derived from in vitro digestion hydrolysate of hen egg white lysozyme. *Food Chem.* **2012**, *135*, 1245-1252.
24. Zheng, L.; Su, G.; Ren, J.; Gu, L.; You, L.; Zhao, M. Isolation and characterization of an oxygen radical absorbance activity peptide from defatted peanut meal hydrolysate and its antioxidant properties. *J. Agric. Food. Chem.* **2012**, *60*, 5431-5437.
25. Dong, Y.; Qi, G.; Yang, Z.; Wang, H.; Wang, S.; Chen, G. Preparation, Separation and Antioxidant Properties of Hydrolysates Derived from Grifola frondosa Protein. *Czech Journal of Food Sciences* **2016**, *33*, 500-506.
26. Chi, C.; Wang, B.; Wang, Y.; Zhang, B.; Deng, S. Isolation and characterization of three antioxidant peptides from protein hydrolysate of bluefin leatherjacket (Navodon septentrionalis) heads. *J. Funct. Foods* **2015**, *12*, 1-10.
27. Zhang, Y.; Liu, J.; Lu, X.; Zhang, H.; Wang, L.; Guo, X.; Qi, X.; Qian, H. Isolation And Identification Of An Antioxidant Peptide Prepared From Fermented Peanut Meal Using Bacillus Subtilis Fermentation. *Int. J. Food Prop.* **2014**, *17*, 1237-1253.
28. Wang, B.; Li, L.; Chi, C.; Ma, J.; Luo, H.; Xu, Y. Purification and characterisation of a novel antioxidant peptide derived from blue mussel (Mytilus edulis) protein hydrolysate. *Food Chem.* **2013**, *138*, 1713-1719.
29. Je, J.; Qian, Z.; Lee, S.H.; Byun, H.; Kim, S. Purification and Antioxidant Properties of Bigeye Tuna (Thunnus obesus) Dark Muscle Peptide on Free Radical-Mediated Oxidative Systems. *J. Med. Food* **2008**, *11*, 629-637.
30. Duan, X.; Ocen, D.; Wu, F.; Li, M.; Yang, N.; Xu, J.; Chen, H.; Huang, L.; Jin, Z.; Xu, X. Purification and characterization of a natural antioxidant peptide from fertilized eggs. *Food Res. Int.* **2014**, *56*, 18-24.
31. Zhang, J.; Zhang, H.; Wang, L.; Guo, X.; Wang, X.; Yao, H. Antioxidant activities of the rice endosperm protein hydrolysate: identification of the active peptide. *Eur. Food Res. Technol.* **2009**, *229*, 709-719.
32. Mendis, E.; Rajapakse, N.; Byun, H.; Kim, S. Investigation of jumbo squid (Dosidicus gigas) skin gelatin peptides for their in vitro antioxidant effects. *Life Sci.* **2005**, *77*, 2166-2178.
33. Je, J.; Park, P.; Kim, S. Antioxidant activity of a peptide isolated from Alaska pollack

(Theragra chalcogramma) frame protein hydrolysate. *Food Res. Int.* **2005**, *38*, 45-50.
